# ﻿The genus *Dryadaula* Meyrick (Lepidoptera, Tineoidea, Dryadaulidae) in China, with descriptions of four new species and a world checklist

**DOI:** 10.3897/zookeys.1074.73067

**Published:** 2021-12-01

**Authors:** Lin-Lin Yang, Hou-Hun Li

**Affiliations:** 1 Institute of Plant Protection, Henan Academy of Agricultural Sciences, Zhengzhou 450002, China Institute of Plant Protection, Henan Academy of Agricultural Sciences Zhengzhou China; 2 College of Life Sciences, Nankai University, Tianjin 300071, China Nankai University Tianjin China

**Keywords:** Asymmetrical genitalia, COI, morphology, taxonomy

## Abstract

Four new species of the genus *Dryadaula* Meyrick, 1893 from China are described: *Dryadaulaauriformis***sp. nov.**, *D.flavostriata***sp. nov.**, *D.hirtiglobosa***sp. nov.** and *D.securiformis***sp. nov.** Photographs of adults and genitalia of the new species are provided. DNA barcodes of *D.auriformis***sp. nov.**, *D.hirtiglobosa***sp. nov.** and *D.securiformis***sp. nov.** are given. A key to the species in China and a detailed checklist for the genus with all 49 known species in the world are presented.

## ﻿Introduction

The family Dryadaulidae was proposed by Regier et al. (2015) on the basis of a molecular phylogenetic study of the Tineoidea. It currently includes two genera: *Dryadaula* Meyrick, 1893 (45 species with worldwide distribution) and *Brachydoxa* Meyrick, 1917 (two species distributed in the Oriental Region). The New Zealand genera *Eschatotypa* Meyrick, 1880 (three species), *Eugennaea* Meyrick, 1915 (one species) and *Sagephora* Meyrick, 1888 (six species) were also considered to belong to this group (Robinson and Nielsen 1993).

The genus *Dryadaula* was established by Meyrick (1893) with *D.glycinopa* Meyrick, 1893 as the type species. It comprises 45 species, distributed in all zoogeographical regions. Most of these species have been placed in subfamilies of Tineidae when originally described. *Dryadaula* was introduced as the senior name of *Thermocrates* Meyrick, 1936 by Robinson (1988) and of *Archimeessia* Zagulajev, 1970, *Chorocosma* Meyrick, 1893, *Cyane* Chambers, 1873, *Choropleca* Durrant, 1914, *Diachalastis* Meyrick, 1920, *Ditrigonophora* Walsingham, 1897, *Opsodoca* Meyrick, 1919 and *Strophalinga* Gozmány & Vári, 1973 by Robinson and Nielsen (1993). The main reason for this situation is that *Dryadaula* is difficult to diagnose externally. However, significant features can be seen when members of *Dryadaula* are dissected: segment VIII and genitalia are strongly modified and asymmetrical in the male, the oviscapt is greatly reduced and the anterior apophyses are rudimentary or absent in the female.

Before this study, only one species *D.epischista* (Meyrick, 1936) in the genus *Dryadaula* was reported from Hong Kong, China (Robinson 1988). We herein describe four new species in China, with illustrations of adults and genitalia and provide a key to the known Chinese species. A world checklist of the genus is also provided to facilitate the further study.

## ﻿Materials and methods

The holotypes of *D.flavostriata* sp. nov. and *D.hirtiglobosa* sp. nov. were collected using sweep nets in the daytime; other type specimens were collected under 250-W high-pressure mercury lamps on white sheets at night. The type specimens are deposited in the Insect Collection, College of Life Sciences, Nankai University, Tianjin, China (**NKU**).

Genitalia dissection and mounting methods follow Li (2002), while head and wing dissections were carried out following the methods described by Lee and Brown (2006). Photographs of the adults were taken with a Leica M205A stereomicroscope, and those of genitalia were taken with a Leica DM750 microscope plus Leica Application Suite 4.6 software. All photographs were refined with Photoshop CS5 software. Protocols for total DNA extraction and mitochondrial COI gene amplification followed that described in our previous study (Yang and Li 2021). Degrees of intra- and interspecific variation of DNA barcode fragments were calculated under the Kimura 2-parameter model using MEGA X. Terminology used in the description of the adult, vein venation and male genitalia follows Robinson and Nielsen (1993) and that of female genitalia follows Regier et al. (2015).

### ﻿Abbreviations used in the text are as follows:

**ANIC**Australian National Insect Collection, CSIRO Division of Entomology, Canberra, Australia;

**BPBM**Bernice Pauahi Bishop Museum, Honolulu, Hawaii, United States of America;

**coll. Baldizzone** collection of Giorgio Baldizzone, Asti Italy;

**coll. Heindel** collection of Richard Heindel, Günzburg, Germany;

**coll. Scholz** collection of Axel Scholz, Illerberg, Germany;

**coll. Sutter** collection of Reinhard Sutter, Bitterfeld, Germany;

**LMNH** Latvian Museum of Natural History, Riga, Latvia;

**MCZ** The Museum of Comparative Zoology, Harvard University, United States of America

**NHM**The Natural History Museum, London, United Kingdom;

**NKU** Insect Collection, College of Life Sciences, Nankai University, Tianjin, China;

**SDEI** Senckenberg Deutsches Entomologisches Institut, Müncheberg, Germany [former: IPE: Institut für Pflanzenschutzforschung, Eberswalde, Germany; and DEI: Deutsches Entomologisches Institut, Deutsche Akademie der Landwirtschaftswissenschaften zu Berlin, Eberswalde, Germany];

**SEL/HNU** Systematic Entomology Laboratory, Hannam University, South Korea;

**TL** Type locality;

**TM**Transvaal Museum, Pretoria, The Republic of South Africa;

**ZIN**Zoological Institute, Russian Academy of Sciences, St. Petersburg, Russia;

**ZMHB** Museum für Naturkunde der Humboldt-Universität (Wolfram Mey), Berlin, Germany;

**ZMUC**Zoological Museum, University of Copenhagen, Denmark.

## ﻿Results

### ﻿DNA Barcoding

The holotype of *Dryadaulaauriformis* sp. nov., a paratype of *D.hirtiglobosa* sp. nov. and two paratypes of *D.securiformis* sp. nov. were successfully sequenced and yielded a barcode of 604 bp. Complementary public sequences of *D.heindeli* Gaedike & Scholz (BOLD:AAL1778, n = 4), *D.terpsichorella* (Busck) (BOLD:AAF9987, n = 139) and *D.visaliella* (Chambers) (BOLD:ACA7671, n = 57; BOLD:AAV6731, n = 17; BOLD:AAV6730, n = 10) from BOLD systems were used to calculate the genetic distance barcode divergence. Sequence divergences are presented in Table 1. The sampled specimens of *D.visaliella* within three Barcode Identification Numbers (BINS) might represent different species, as members of them show higher divergences from each other and were not well distinguished by barcodes.

**Table 1.** Percentage of divergence in the cytochrome c oxidase subunit I (COI) gene sequences of the *Dryadaula* species.

	1	2	3	4	5	6	7	8
1 *D.auriformis* sp. nov.	–	–	–	–	–	–	–	–
2 *D.hirtiglobosa* sp. nov.	18.55	–	–	–	–	–	–	–
3 *D.securiformis* sp. nov.	19.18	6.06	**0**	–	–	–	–	–
4 *D.heindeli*	17.74–17.95	13.61–13.81	14.31–14.81	**0–0.17**	–	–	–	–
5 *D.terpsichorella*	18.64–19.83	10.41–12.05	9.97–11.92	9.97–10.73	**0–0.35**	–	–	–
6 *D.visaliella* (ACA7671)	15.20–21.36	9.33–10.79	10.00–12.79	11.04–13.85	9.33–12.49	**0–1.98**	–	–
7 *D.visaliella* (AAV6731)	18.74–22.70	10.33–11.26	10.62–11.64	15.29–18.58	11.12–14.49	**3.34–13.27**	**0–0.51**	–
8 *D.visaliella* (AAV6730)	20.57–21.22	9.20–9.62	10.22–10.99	13.18–14.43	10.19–11.89	**6.93–9.65**	**11.51–13.67**	**0–0.34**

Genetic distances (%) were corrected with the Kimura two-parameter (K2P) substitution model using MEGA X; extreme values of intraspecific and interspecific distances are given (the numbers in bold are the intraspecific distances).

### ﻿Taxonomic accounts

#### ﻿ Dryadaula

Taxon classificationAnimaliaLepidopteraTineidae

Meyrick, 1893

E3D3E547-FFA2-55C2-8A4D-D65FE4B78691

Dryadaula Meyrick, 1893: 559. Type species: Dryadaulaglycinopa Meyrick, 1893, by monotypy. TL: Australia (New South Wales).
Cyane Chambers, 1873: 112. Synonymised by Robinson and Nielsen 1993: 55. Type species: Cyanevisaliella Chambers, 1873, by monotypy. TL: United States (Kentucky).
Chorocosma Meyrick, 1893: 560. Synonymised by Robinson and Nielsen 1993: 55. Type species: Chorocosmamelanorma Meyrick, 1893, by monotypy. TL: Australia (Sydney).
Ditrigonophora Walsingham, 1897: 117. Synonymised by Robinson and Nielsen 1993: 55. Type species: Ditrigonophoramarmoreipennis Walsingham, 1897, by original designation. TL: Grenada (Balthazar).
Choropleca Durrant, 1914: 366. Objective replacement name for Cyane Chambers, 1873. Synonymised by Robinson and Nielsen 1993: 55.
Opsodoca Meyrick, 1919: 270. Synonymised by Robinson and Nielsen 1993: 55. Type species: Opsodocametrodoxa Meyrick, 1919, by original designation. TL: Guyana.
Diachalastis Meyrick, 1920: 363. Synonymised as Choropleca Durrant by Clarke, 1971: 221. Synonymised by Robinson and Nielsen 1993: 55. Type species: Diachalastistetraglossa Meyrick, 1920, by monotypy. TL: Fiji.
Thermocrates Meyrick, 1936: 620. Synonymised by Robinson, 1988: 74. Type species: Thermocratesepischista Meyrick, 1936, by monotypy. TL: Japan (Kyushu).
Archimeessia Zagulajev, 1970: 658. Synonymised by Robinson and Nielsen 1993: 55. Type species: Archimeessiazinica Zagulajev, 1970, by original designation. TL: Azerbaijan.
Strophalinga Gozmány & Vári, 1973: 9. Synonymised by Robinson and Nielsen 1993: 55. Type species: Tineaglycinocoma Merick, 1932, by original designation. TL: Ethiopia.

##### Diagnosis.

**Figures 1–4.**
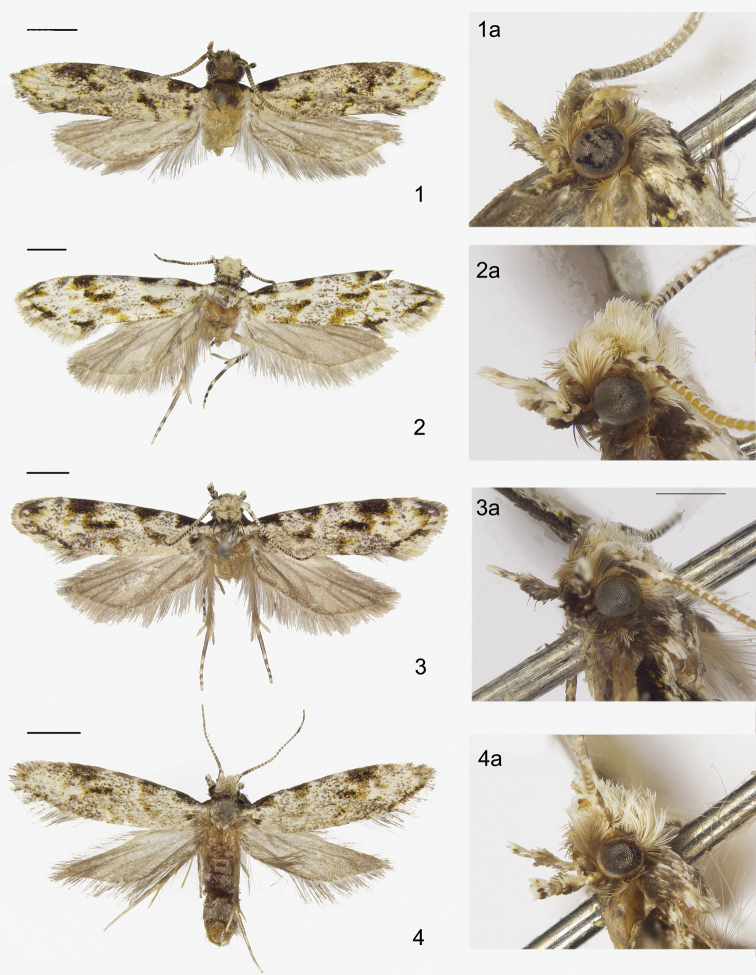
Adults of *Dryadaula* species **1***Dryadaulaauriformis* sp. nov., male holotype **1a** lateral view of head **2***D.flavostriata* sp. nov., male holotype **2a** lateral view of head **3***D.hirtiglobosa* sp. nov., male holotype **3a** lateral view of head **4***D.securiformis* sp. nov., male holotype **4a** lateral view of head. Scale bars: 1.0 mm.

**Figures 5, 6.**
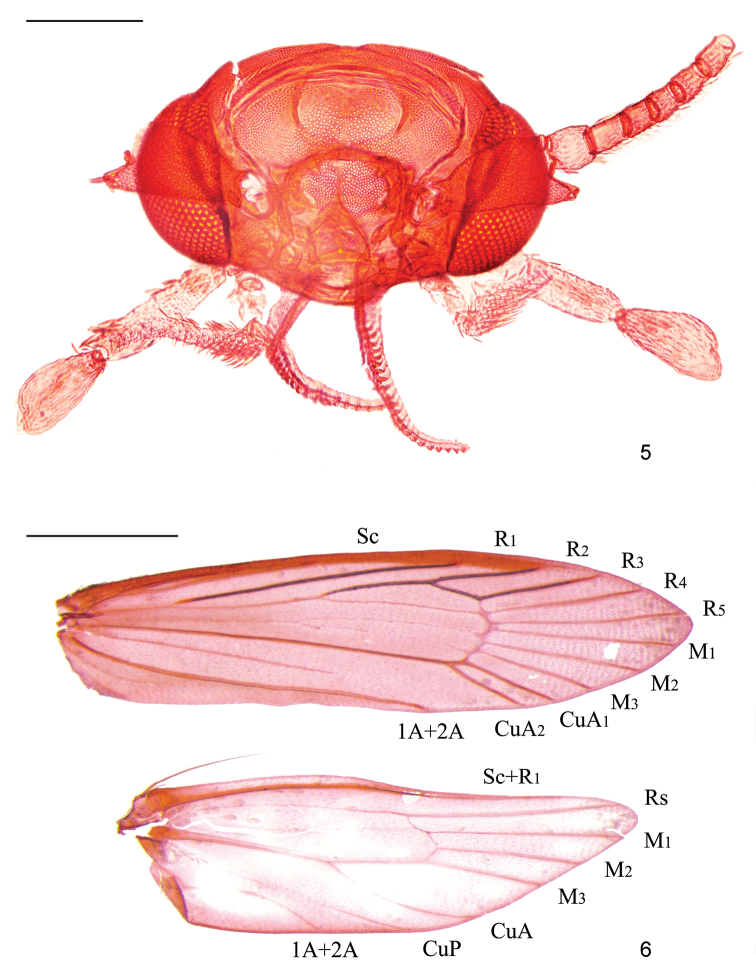
*Dryadaulasecuriformis* sp. nov., male paratype, slide No. DNAYLL18121 **5** head **6** Wing venation. Scale bars: 0.2 mm (head); 1.0 mm (wing venation).

**Figures 7, 8.**
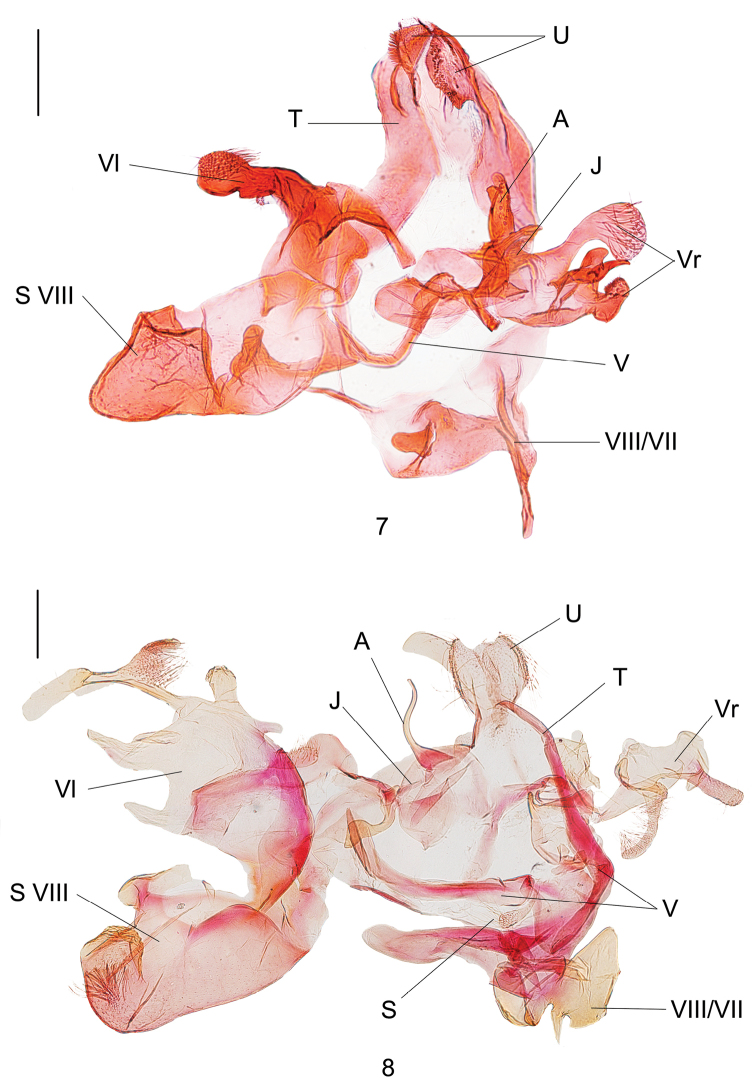
Male genitalia of *Dryadaula* species **7***D.auriformis* sp. nov., holotype, slide No. DNAYLL18124 **8***D.flavostriata* sp. nov., holotype, slide No. YLL11112. Scale bars: 0.25 mm. (U = uncus lobes; T = tegumen; V = vinculum; S = saccus; S VIII = sternum VIII; Vr = right valva; Vl = left valva; J = juxta; A = aedeagus).

**Figures 9, 10.**
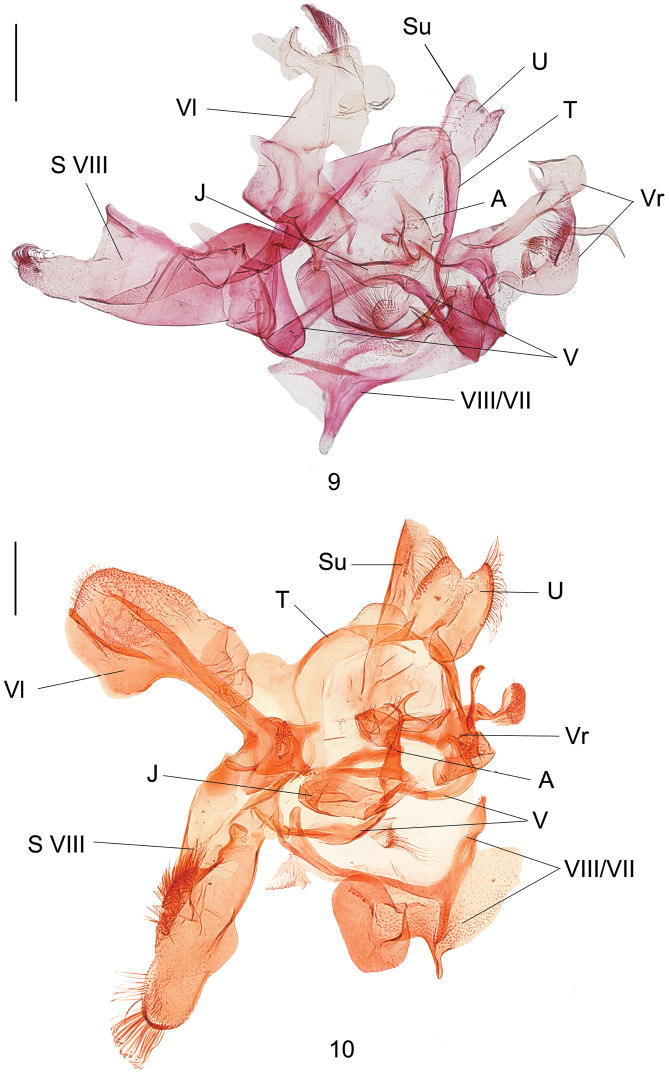
Male genitalia of *Dryadaula* species **9***D.hirtiglobosa* sp. nov., holotype, slide No. YLL13026 **10***D.securiformis* sp. nov., paratype, slide No. DNAYLL18173. Scale bars: 0.25 mm. (U = uncus lobes; T = tegumen; V = vinculum; Su = Subscaphium ; S VIII = sternum VIII; Vr = right valva; Vl = left valva; J = juxta; A = aedeagus).

**Figure 11.**
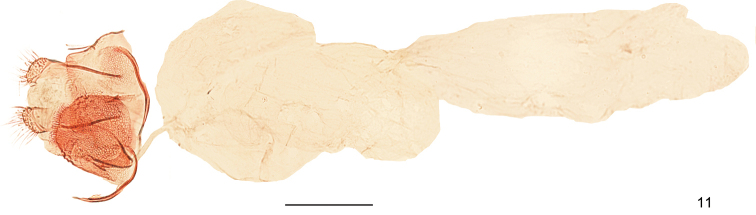
Female genitalia of *Dryadaulasecuriformis* sp. nov., paratype, slide No. DNAYLL18123. Scale bars: 0.25 mm.

*Dryadaula* is a small-sized moth, with wingspans of no more than 20 mm. It can be recognised by the following characters: head (Figs 1a–4a, 5) with erect piliform scales, transfrontal suture inverted V-shaped; antennae 0.7× length of forewing, scape without pecten, flagellomeres with single annulus or two annuli of contrasting-coloured scales; labial palpus spatulate, bearing lateral bristles; forewing often brightly coloured, with venation (Fig. 6) complete, CuP weak; hind-wing with M_3_ or CuA_1_ absent; female with single frenulum bristle; segment VIII reduced and highly modified, usually asymmetrical in male; male genitalia (Figs 7–10) strongly asymmetrical, incorporating part of sternum VII and sternum VIII; aedeagus fused with right valva; gnathos absent; uncus lobes usually fused; female (Fig. 11) oviscapt reduced, posterior apophyses short, anterior apophyses rudimentary or absent, sternum VIII hardly developed.

##### Distribution.

Worldwide; the distribution of each species is given in Table 2.

**Table 2.** World checklist of the genus *Dryadaula* Meyrick, 1893.

	Species	Distribution	Depository of type
1	*acrodisca* (Meyrick, 1917): 79. (*Choropleca*)	Guyana	NHM (LT)
	TL: Guyana (Mallali).		
2	*amentata* (Meyrick, 1919): 271. (*Opsodoca*)	Guyana	NHM (HT)
	TL: Guyana (Bartica). Figs: Clarke (1970: pl. 34, fig. 2 adult and male genitalia).		
3	*advena* (Zimmerman, 1978): 326. (*Choropleca*)	United States	BMH (HT)
	TL: United States (Hawaii). Figs: Zimmerman (1978: fig. 156-A male genitalia; fig. 481-A adult).		
4	*anthracorma* Meyrick, 1915: 369.	Australia	NHM (LT and PLT)
	TL: Australia (Victoria). Figs: Robinson and Nielsen (1993: fig. 63 adult; fig. 70 male genitalia; figs 71, 72 female genitalia); Robinson (2009: fig. 2 adult).		
5	*auriformis* sp. nov.	China	NKU (HT and PT)
	TL: China (Hainan). Figs 1, 7.		
6	*boviceps* (Walsingham, 1914): 366. (*Choropleca*)	Mexico	NHM (HT)
	TL: Mexico (Guerrero). Figs: Robinson (2009: fig. 4 adult).		
7	*brontoctypa* (Meyrick, 1880): 259. (*Ereunetis*)	Australia	NHM (LT and PLT)
	TL: Australia (Sydney).		
8	*castanea* Philpott, 1915: 201.	New Zealand	?
	TL: New Zealand (Bluff, Invercargill).		
9	*catorthota* (Meyrick, 1917): 80. (*Choropleca*)	Guyana	NHM (LT and PLT)
	TL: Guyana (Mallali).		
10	*caucasica* (Zagulajev, 1970): 662. (*Archimeessia*)	Azerbaijan, Poland, Russia, Sweden	ZIN (HT)
	TL: Azerbaijan (Artschevan). Figs: Zagulajev (1970: fig. 6 female genitalia); Zagulajev (1979: fig. 64 adult; fig. 65 female genitalia); Sachkov (1995: fig. 7 male genitalia); Jaworski et al. (2012: fig. 1 adult); Gaedike (2015: pl. 1, fig. 1 adult; drawings, male genitalia 1; drawings, female genitalia 1).		
11	*discatella* (Walker, 1864): 1021. (*Gelechia*)	Brazil	NHM (HT)
	TL: Brazil.		
12	*epischista* (Meyrick, 1936): 621. (*Thermocrates*)	China (Hong Kong), Japan	NHM (HT)
	TL: Japan (Kyushu). Figs: Robinson (1988: fig. 1 adult; fig. 2 abdominal pelt; figs 3, 4 male genitalia); Sakai (2013: fig. 3–12–13 adult).		
13	*epixantha* (Turner, 1923): 184. (*Erechthias*)	Australia	ANIC
	TL: Australia (Queensland).		
14	*flavostriata* sp. nov.	China	NKU (HT)
	TL: China (Guangxi). Figs 2, 8.		
15	*germana* (Walsingham, 1914): 367. (*Choropleca*)	Mexico	NHM (HT)
	TL: Mexico (Guerrero).		
16	*glycinocoma* (Meyrick, 1932): 120. (*Tinea*)	Ethiopia	NHM (LT and PLT)
	TL: Ethiopia. Figs: Gozmány and Vári (1973: fig. 6 male genitalia).		
17	*glycinopa* Meyrick, 1893: 559.	Australia	NHM (LT)
	TL: Australia (New South Wales). Figs: Robinson and Nielsen (1993: fig. 64 adult; fig. 136 wing venation).		
18	*heindeli* Gaedike & Scholz, 1998: 106.	Belgium, France, Germany, Italy, Netherlands, Norway, Spain, Switzerland	SDEI (HT and PT); coll. Scholz (PT); coll. Heindel (PT); coll. Sutter (PT)
	TL: Germany (Bayem). Figs: Gaedike and Scholz (1998: fig. 1 adult; figs 3–6 male genitalia; fig. 7 female genitalia; figs 10–15 larva, chaetotaxy and pupa); Gaedike (2015: pl. 1, fig. 5 adult; drawings, male genitalia 5; drawings, female genitalia 5).		
19	*hellenica* (Gaedike, 1988): 331. (*Archimeessia*)	Greece	ZMUC (HT and PT); SDEI (PT).
	TL: Greece (Peloponnese). Figs: Gaedike (1988: figs 22–26 male genitalia); Gaedike (2015: pl. 1, fig. 7 adult; drawings, male genitalia 7; drawings, female genitalia 7).		
20	*hirtiglobosa* sp. nov.	China	NKU (HT and PT)
	TL: China (Guangxi). Figs 3, 9.		
21	*irinae* (Savenkov, 1989): 94. (*Archimeessia*)	Austria, Bulgaria, Latvia, Poland, Slovakia,	LMNH
	TL: Latvia. Figs: Savenkov (1989: figs. 1–3); Pastorális et al. (2011: figs 1, 2 adults; fig. 3 male genitalia); Jaworski et al. (2014: fig. 5 adult; fig. 6 larva; fig. 7 larval shelter; fig. 8 pupal case); Gaedike (2015: pl. 1, fig. 3 adult; drawings, male genitalia 3; drawings, female genitalia 3).		
22	*isodisca* (Meyrick, 1917): 80. (*Choropleca*)	Guyana	NHM (LT)
	TL: Guyana (Bartica, Mallali).		
23	*koreana* Roh & Byun, 2020: 222	South Korea	SEL/HNU (HT and PT)
	TL: South Korea (Jeollanam-do). Figs: Roh et al. (2020: figs. 1, 2 adult; fig. 3: wing venation; fig. 4 male genitalia; fig. 5 female genitalia).		
24	*marmoreipennis* (Walsingham, 1897): 118. (*Ditrigonophora*)	Grenada	NHM (LT)
	TL: Grenada (Balthazar).		
25	*melanorma* (Meyrick, 1893): 560. (*Chorocosma*)	Australia	NHM (HT)
	TL: Australia (Sydney).		
26	*mesosticha* (Turner, 1923): 184. (*Erechthias*)	Australia	ANIC
	TL: Australia (Queensland).		
27	*metrodoxa* (Meyrick, 1919): 271. (*Opsodoca*)	Guyana	NHM (LT)
	TL: Guyana (Bartica, Mallali). Figs: Clarke (1970: pl. 34, fig. 1 adult, wing venation and male genitalia).		
28	*minuta* Gaedike, 2007: 160.	Greece, Turkey	ZMHB (HT), ZMUC (PT)
	TL: Turkey (Mugla). Figs: Gaedike (2007: fig. 1 adult; figs 12, 13 male genitalia; fig. 14 female genitalia); Gaedike (2015: pl. 1, fig. 4 adult; drawings, male genitalia 4; drawings, female genitalia 4).		
29	*multifurcata* Gaedike, 2000: 358.	Russia	ZIN (HT and PT)
	TL: Russia (Primorskij kraj). Figs: Gaedike (2000: figs 5–7 male genitalia; fig. 8 female genitalia).		
30	*murenula* (Meyrick, 1924): 65. (*Choropleca*)	Peru	NHM (LT and PLT)
	TL: Peru (Jurimaguas, Iquitos).		
31	*myrrhina* Meyrick, 1905: 243.	New Zealand	NHM (HT)
	TL: New Zealand. Robinson (2009: fig. 3 adult).		
32	*napaea* Meyrick, 1905: 244.	Australia	NHM (HT)
	TL: Australia (Tasmania).		
33	*nedae* (Gaedike, 1983): 125. (*Infurcitinea*)	Croatia, Cyprus, Greece, Turkey	coll. Baldizzone (HT and PT); SDEI (PT)
	TL: Greece. Figs: Baldizzone (1983: figs 3–7 male genitalia; fig. 8 female genitalia); Gaedike (2015: pl. 1, fig. 8 adult; drawings, male genitalia 8; drawings, female genitalia 8).		
34	*pactolia* Meyrick, 1901: 577.	Denmark, France, Germany, Great Britain, New Zealand, Netherlands, Portugal, Switzerland.	NHM (LT)
	TL: New Zealand. Figs: Gaedike and Scholz (1998: fig. 2 adult; figs 8, 9 male genitalia); Gaedike (2015: pl. 1, fig. 6 adult; drawings, male genitalia 6; drawings, female genitalia 6).		
35	*panscia* Meyrick, 1917: 81. (*Choropleca*)	Ecuador	NHM (LT)
	TL: Ecuador (Huigra).		
36	*placens* Meyrick, 1920: 363. (*Hectacma*)	Australia	NHM (HT)
	TL: Australia (Queensland).		
37	*poecilta* Walsingham, 1914: 366. (*Choropleca*)	Mexico	NHM (HT)
	TL: Mexico (Guerrero).		
38	*rhombifera* Meyrick, 1917: 82. (*Choropleca*)	Guyana	NHM (HT)
	TL: Guyana (Mallali).		
39	*securiformis* sp. nov.	China	NKU (HT and PT)
	TL: China (Hainan). Figs 4, 5, 6, 10 and 11.		
40	*selenophanes* (Meyrick, 1880): 259. (*Ereunetis*)	Australia	NHM (LT)
	TL: Australia (Queensland).		
41	*sublimis* (Meyrick, 1917): 81. (*Choropleca*)	Colombia	NHM (LT)
	TL: Colombia (La Crumbre).		
42	*terpsichorella* (Busck, 1910): 134. (*Cyane*)	Fiji, Hawaii, Rapa.	NHM (HT)
	TL: Hawaii (Honolulu). Figs: Clarke (1971: pl. 28, figs. a, b adult; fig. 172 male genitalia, coremata and 8^th^ segment; fig. 173 wing venation and female genitalia); Zimmerman (1978: fig. 157 head and wing venation; fig. 159 adult, male and female genitalia; fig. 160 abdomen and male genitalia); Robinson (2009: fig. 5 adult).		
43	*trapezoides* (Meyrick, 1935): 579. (*Tinea*)	Japan	NHM (LT)
	TL: Japan (Tokyo). Figs: Sakai (2013: figs 3–12–14 adult; fig. Tin12 female genitalia).		
44	*tripudians* (Meyrick, 1924: 65). (*Choropleca*)	Peru	NHM (LT)
	TL: Peru (Jurimaguas).		
45	*ussurica* Gaedike, 2000: 358.	Russia	ZIN (HT and PT)
	TL: Russia (Primorskij kraj). Figs: Gaedike (2000: figs. 1–3 male genitalia; fig. 4 female genitalia).		
46	*visaliella* (Chambers, 1873): 113. (*Cyane*)	Canada, United States	MCZ (ST)
	TL: United States (Kentucky). Figs: Zimmerman (1978: fig. 158 wing venation); Regier et al. (2015: fig. 6F adult; fig. 10 head, wing venation, male and female genitalia).		
47	*zinica* (Zagulajev, 1970: 661). (*Archimeesia*)	Azerbaijan, Russia	ZIN (HT and PT); NHM (PT)
	TL: Azerbaijan. Figs: Zagulajev (1970: fig. 1 head; fig. 2 wing venation; fig. 3 legs; fig. 4 male genitalia; fig. 5 female genitalia); Gaedike (2015: pl. 1, fig. 2 adult; drawings, male genitalia 2; drawings, female genitalia 2).		
48	*zygodes* Meyrick, 1918: 44. (*Tinea*)	South Africa	TM (HT)
	TL: South Africa (Natal). Figs: Janse (1968: pl. 69, fig. 3 adult, fig. 4 male genitalia; pl. 109: fig. 7 wing venation; pl. 111, fig. 16 labial palpi and maxillary palpi; pl. 113, fig. 9 male genitalia); Gozmány and Vári (1973: fig. 58 male genitalia)		
49	*zygoterma* Meyrick, 1917: 82. (*Choropleca*)	Colombia, Ecuador	NHM (LT)
	TL: Colombia (La Crumbre).		

##### Biology.

The larvae of some species are detritivores or feed on lichens and fungi. The biology of *Dryadaula* was reviewed or summarised by Robinson and Nielsen (1993), Gaedike (2015) and Regier et al. (2015).

### ﻿Key to Chinese *Dryadaula* species, based on the male

**Table:** 

1	Forewing dull ochreous brown patterned with yellow-brown and white streaks (Robinson 1988: fig. 1)	***D.epischista***
–	Forewing white patterned with black spots or patches	**2**
2	Subscaphium absent	**3**
–	Subscaphium present	**4**
3	Uncus lobes without process; vinculum without additional lobe; left valva clavate; right valva with dorsal lobe globular apically, ventral lobe with three prominences (Fig. 7)	***D.auriformis* sp. nov.**
–	Uncus lobes with a rectangular process; vinculum with a lobe; left valva sub-oval; right valva with dorsal lobe vaulted, having a finger-like process apically, ventral lobe digitate, without prominence (Fig. 8)	***D.flavostriata* sp. nov.**
4	Modification attached to vinculum is receptacle-shaped, with a sharp horn and a drumstick-like process; left valva irregular in shape; right valva with dorsal lobe having a subquadrate and a digitate process, ventral lobe crescent; juxta pocket-like (Fig. 9)	***D.hirtiglobosa* sp. nov.**
–	Modification attached to vinculum comprising of a Y-shaped sclerotisation and a receptacle-shaped sclerite; left valva battle axe-shaped; right valva with dorsal and ventral lobes slender, S-shaped; juxta elliptical (Fig. 10)	***D.securiformis* sp. nov.**

#### ﻿ Dryadaula auriformis sp. nov.

Taxon classificationAnimaliaLepidopteraTineidae

CC46BA4C-D85A-50E9-A9BF-07C1FA1A5205

http://zoobank.org/816AD20C-6E06-4A79-885F-9B8A00F93290

Figures 1 , 7

##### Type material.

***Holotype***: China: • ♂; Hainan Province, Mt. Jianfeng (18°44'N, 108°52'E); alt. 787 m; 1.vi.2015; leg. Peixin Cong; genitalia slide No. DNAYLL18124. ***Paratype***: China: • 1 ♂; Hainan Province, Mt. Jianfeng; alt. 745 m; leg. Xia Bai; genitalia slide No. XMR18217.

##### Differential diagnosis.

The new species is externally similar to *D.zinica* (Zagulajev, 1970), but can be separated from it by the male genitalia structures. In *D.auriformis* sp. nov., the left valva is narrowed and clavate, the right valva is bilobate, the bullet-like sternum VIII is smooth in the male genitalia, whereas in *D.zinica*, the left valva is broad, the right valva is not divided and the sternum VIII bears long and thin bristles on outer margin.

##### Description.

**Adult** (Fig. 1): Wingspan 8.5 mm in holotype, 9.0 mm in paratype. Vertex and frons smoky grey, tinged with black scales anterior of antenna. Antenna with scape white, except for a black spot at dorsal base; flagellum with alternate yellowish-white and cinereous annulations, cinereous on dorsal surface of basal 2–4 flagellomeres, with three narrow cinereous bands towards apex. Labial palpus spatulate; yellowish-white, first palpomere and basal 3/4 of second palpomere black on outer surface, third palpomere black at base on inner surface, with three black dots on outer surface. Thorax and tegula blackish-brown in anterior 1/2, white in posterior 1/2. Forewing ground colour white, irrorate with blackish-brown scales, edged with bright ochreous yellow scales along of termen and markings; patterned with black markings: costa with a wedge-shaped spot at base, a rectangular spot at 2/5, an obscure dot at middle, an oval patch from 3/5 to 4/5; cell with an obscure irregular spot at distal 1/4, tending to coalesce with oval costal patch; fold with irregular stripes at base, basal 1/3 and 2/3, obliquely inward towards dorsum; an interrupted terminal line around apex then along termen to tornus; cilia white in basal 1/2, grey in distal 1/2, with individual scales dark-tipped. Hind-wing and cilia grey. Legs greyish-white, tibia black on outer surface, tarsus black on dorsal surface, except for end of each tarsomere.

***Male genitalia*** (Fig. 7). Uncus lobes small, ear-shaped, bearing dense setae dorso-apically. Subscaphium not developed. Tegumen somewhat broad. Vinculum narrowed, deeply arched at middle, without additional lobe. Saccus not developed. A complicated, irregular, sclerotised modification attached to vinculum anteriorly, possibly part of segments VII and VIII; its left part rectangular, with a stout digitation, its right part stem-like. Sternum VIII articulated with vinculum at left, articulated with left valva dorso-basally; somewhat bullet-like, narrowly rounded and folded apically, triangularly folded at 1/3 on ventral margin. Valvae strongly asymmetrical. Left valva clavate, bent outwards; its basal part skirt-like, arched anteriorly, distal part a globular, setose lobe, a small, digital, setose lobe at distal 1/3. Right valva bilobate: dorsal lobe with a thumb-like process articulated with juxta at base, middle part curved like a gooseneck, distal part globular, setose; ventral lobe with three prominences, one stout, finger-like, one slightly twisted, horn-shaped and one hammer-shaped. Juxta irregular in shape. Aedeagus a curved horn with a stout base; cornutus absent.

**Female.** Unknown.

##### Distribution.

China (Hainan).

##### Etymology.

The specific name is derived from the Latin *auriformis*, meaning ear-shaped, referring to the ear-shaped uncus lobes.

##### DNA barcode.

One DNA barcode from the holotype was generated and deposited in GenBank and BOLD systems: MZ711361/ DRYAD001-21. *Dryadaulaauriformis* sp. nov. is clearly distinguishable by its DNA barcode from its congeners, the minimum divergence (Table 1) to the nearest species, *D.heindeli* Gaedike & Scholz, is 17.74–17.95%.

#### ﻿ Dryadaula flavostriata sp. nov.

Taxon classificationAnimaliaLepidopteraTineidae

01ECDD30-9C3D-512B-892C-B3EDA7737A1D

http://zoobank.org/59375061-706B-4229-A4C2-B919D4DA48AB

Figures 2 , 8

##### Type material.

***Holotype***: China: • ♂; Guangxi Province, Nanning City, Mt. Daming (23°24'N, 108°30'E); alt. 1250 m; 23.v.2011; leg. Linlin Yang & Yinghui Mou; genitalia slide No. YLL11112.

##### Differential diagnosis.

*Dryadaulaflavostriata* sp. nov. is similar to *D.caucasica* (Zagulajev, 1970), but differs from it by the forewing peppered with more ochreous yellow scales that form stripes between fold and dorsum; the male genitalia with short uncus that is equipped with a rectangular process at the left and the sub-oval left valva with processes of different shapes, not bearing thorns or long bristles. In *D.caucasica*, the forewing has dark grey-brown longitudinal stripes between fold and dorsum; in the male genitalia, the elongate uncus has no process and the fluted left valva has dense, long bristles along outer ventral margin and a globular sclerotisation which is densely thorned.

##### Description.

**Adult** (Fig. 2): Wingspan 11.5 mm in holotype. Vertex and frons yellowish-white. Antenna with scape brightly white, margined with black anteriorly; flagellum with dorsal surface alternating black and white in basal 1/3, black in medial 1/3, alternating black and white every two annuli in distal 1/3, ventral surface white in basal 2/3, alternating black and white every two annuli in distal 1/3. Labial palpus spatulate; first palpomere black, second palpomere white on inner surface, black on outer surface; third palpomere white tinged ochreous yellow, with a black dot at middle of outer surface. Thorax and tegula blackish-brown in anterior 1/2, brightly white tinged with blackish-brown in posterior 1/2. Forewing brightly white, with scattered grey and black scales; patterned with black markings that are bordered with ochreous yellow and ochreous yellow markings that are tinged with black: costa with a wedge-shaped spot at base, a semicircular spot at 1/3, a semicircular patch at 3/4 and an arc line at apex, diffused greyish-black smudges amongst spots; cell with a narrowed, oval spot at distal 1/4, suffused with ochreous yellow anterolaterally, tending to coalesce with semicircular costal patch; fold with irregular ochreous yellow stripes at base, basal 1/3 and 2/3, tinged with black, obliquely inwards towards dorsum; four or five black dots from apex to tornus along termen, forming a broken terminal line; cilia white in basal 1/2, grey in distal 1/2. Hind-wing and cilia dark grey. Foreleg dark grey, tarsus yellowish-white on inner surface; mid-leg femur greyish-black on outer surface, pale yellow on inner surface, tibia and tarsomeres yellowish-white at end; hind-leg yellowish-white on inner and ventral surface, tibia grey on outer and dorsal surface, tarsus greyish-black on outer and dorsal surface, yellowish-white at end of each tarsomere.

***Male genitalia*** (Fig. 8). Uncus lobes fused into a shield, bearing long hairs dorsally, slightly concave at middle on posterior margin, asymmetrical, left lobe slightly longer than right, with a smooth rectangular process. Subscaphium not developed. Tegumen simple, forming a narrow ring with vinculum. Vinculum broad, posterior margin strongly sclerotised, with a setose lobe at right. Saccus subtriangular, asymmetrical. A complicated, irregular, sclerotised modification attached to vinculum anteriorly, possibly part of segments VII and VIII; its left part somewhat triangular, right part chestnut-shaped. Sternum VIII articulated with vinculum at left, articulated with left valva dorso-basally, strongly modified into a large pocket, broadly rounded apically, with a large hammer-like process at middle, with a tuft of non-deciduous hairs. Valvae strongly asymmetrical. Left valva larger than right one, sub-oval; costal margin with a broad, vertical bridge at middle, a digitate process at distal 1/3, a larger subrectangular process at 1/6, a slender, curved process at end that is about 2/3 length of valva, with a setose fan-shaped lobe at middle; ventral margin with an oblique, digitate process at middle and a horned process at end. Right valva divided into two parts: dorsal lobe large, vaulted, with a finger-like process apically and a slender, decurved, setose lobe at middle; ventral lobe digitate, hooked apically. Juxta inflated and rounded, with a U-shaped process on left, an arced band on right. Aedeagus as long as saccus, simple, expanded in basal 1/4, sinuate in S-shape in distal 3/4; cornutus absent.

**Female.** Unknown.

##### Distribution.

China (Guangxi).

##### Etymology.

The specific name is derived from the Latin prefix *flav*-, meaning yellowish and the Latin word *striatus*, stripe, referring to the forewing with inwardly oblique ochreous yellow stripes between fold and dorsum.

#### ﻿ Dryadaula hirtiglobosa sp. nov.

Taxon classificationAnimaliaLepidopteraTineidae

6E709E13-5D64-5D76-AD2E-454A7140D977

http://zoobank.org/3BC5ADCC-97DA-4685-B9C4-39D6C013B943

Figures 3 , 9

##### Type material.

***Holotype***: China: • ♂; Guangxi Province, Nanning City, Mt. Daming (23°24'N, 108°30'E); alt. 1250 m; 23.v.2011; leg. Linlin Yang & Yinghui Mou; genitalia slide No. YLL13026. ***Paratypes***: China: • 1 ♂; Zhejiang Province, Jingning She Autonomous County, Wangdongyang Wetland Reserve (27°24'N, 119°23'E); alt. 1174 m; 16.viii.2018; leg. Shuai Yu et al.; genitalia slide No. DNAYLL18170 • 1 ♂; Zhejiang Province, Jiangshan City, Mt. Xianxia, Shuangxikou Town, Laofoyan Village (28°22'N, 118°40'E); alt. 400 m; 26.v.2017; leg. Shuonan Qian and Jiaen Li; genitalia slide No. DNAYLL18169.

##### Differential diagnosis.

The new species is externally close to *D.irinae* (Savenkov, 1989), but differs from it by the shape of valva in the male genitalia: in *D.hirtiglobosa* sp. nov., the left valva has a blade-shaped setose lobe apically and a sword hilt-like process subapically on ventral margin and the right valva is bilobate; in *D.irinae*, the left valva is divided into two parts, without blade-shaped setose lobe or sword hilt-like process, the right valva is not bilobate.

##### Description.

**Adult** (Fig. 3): Wingspan 11.0 mm in holotype, 9.5–10.5 mm in paratypes. Vertex cream white, frons pale greyish-brown. Antenna with scape brightly white, margined with black anteriorly; flagellum with ventral surface bearing white scales on alternate annuli, dorsal surface alternating black and white in basal 2/3, white with three black bands in distal 1/3. Labial palpus spatulate; smoky white on inner surface, black on outer surface, except for yellowish-white at end of third palpomere. Thorax and tegula blackish-brown in anterior 1/2, white tinged with blackish-brown in posterior 1/2. Forewing white, with scattered black and brown scales, patterned with black markings that are edged with ochreous yellow: costa with a wedge-shaped stripe in basal 1/6, a semicircular spot at 1/3, an ambiguous dot at middle, a semicircular patch at 3/4, an obscure, irregular greyish fuscous dot near apex and a dot at apex; cell with a narrowed, oval spot at distal 1/3, more or less coalesced with semicircular costal patch; fold with irregular stripes at base, basal 1/3 and 2/3, powdered with ochreous yellow scales, obliquely inwards towards dorsum; terminal line black, not continuous, around apex, then along termen to tornus; cilia greyish-white, with some black dots. Hind-wing and cilia dark grey. Foreleg greyish-black, tarsomeres white at end; mid-leg femur greyish-black on outer surface, pale yellow on inner surface, tarsomeres yellowish-white at end; hind leg with tibia yellowish-white on inner and ventral surface, grey on outer and dorsal surface, tarsomeres yellowish-white on inner and ventral surface, greyish-black on outer and dorsal surface, brightly white at end.

***Male genitalia*** (Fig. 9). Uncus lobes fused into a shield, bearing long hairs, slightly concave at middle on posterior margin, asymmetrical. Subscaphium ribbon-like. Tegumen asymmetrical, left part broader than right part, forming a narrow ring with vinculum. Vinculum arched, narrowed, equipped with a globular lobe bearing long hairs at middle. Saccus not developed. A receptacle-shaped, sclerotised modification attached to vinculum anteriorly, possibly part of segments VII and VIII, with a sharp horn and a drumstick-like process at right on posterior margin. Sternum VIII articulated with vinculum at left, articulated with left valva dorso-basally; strongly modified, folded, concave at middle on costal margin, convex in hillock shape on ventral margin; broad in basal 3/4, with a digitate basal process, narrowed and thumb-like in distal 1/4, bearing long scales apically. Valvae strongly asymmetrical. Left valva larger than right one, irregular in shape, with a blade-shaped setose lobe apically; costal margin with a triangular protuberance at base and a 1/2-round protuberance near apex; ventral margin with a sword-hilt-like process subapically; inner surface with a finger-like lobe at base, a small, subquadrate, smooth process at middle near costal margin and a hillock-shaped smooth process near apex; apodeme distinct. Right valva divided into two parts: dorsal lobe strongly sclerotised, expanded and convex dorso-apically, with a large subquadrate and a small digitate process, with a vaulted, setose lobe ventro-apically; ventral lobe crescent, with a slender, hooked process distally, a membranous, hillock-shaped process that bears dense setae at middle of costal margin, a horned process at base of inner surface. Juxta broad, pocket-like, with a curved thorn-like process at end of right side. Aedeagus short, as long as modification of vinculum, slightly expanded and membranous basally; sclerotised near lateral sides, with a small process on right; tapered to pointed apex; cornutus absent.

**Female.** Unknown.

##### Distribution.

China (Guangxi, Zhejiang).

##### Etymology.

The specific name is derived from the Latin prefix *hirt*-, from *hirtus* meaning hairy and the Latin word *globosus*, globular, referring to the vinculum equipped with a globular lobe bearing long hairs.

##### DNA barcode.

One DNA barcode from a paratype was generated and deposited in GenBank and BOLD systems: MZ711362/ DRYAD002-21. The minimum divergence (Table 1) to its nearest species, *D.securiformis* sp. nov., is 6.06%.

#### ﻿ Dryadaula securiformis sp. nov.

Taxon classificationAnimaliaLepidopteraTineidae

18A64CC6-3505-5425-B06C-DB43603C1F37

http://zoobank.org/14EFF095-5E6A-4C29-8513-243C3B127E8F

Figures 4 , 5–6 , 10 , 11

##### Type material.

***Holotype***: China: • ♂; Hainan Province, Mt. Jianfeng (18°44'N, 108°52'E); alt. 787 m; 5.iii.2016; leg. Qingyun Wang. ***Paratypes***: China: • 8♂, 1♀; same data as holotype, except dated 4–8.iii.2016; genitalia slide Nos DNAYLL18121m, DNAYLL18122m, DNAYLL18172, DNAYLL18173, XMR18158, XMR18334, XMR18335 • 1♂; Hainan Province, Mt. Jianfeng; alt. 770 m; 29.v.2015; leg. Peixin Cong; genitalia slide No. DNAYLL18171 • 2♀; Hainan Province, Mt. Jianfeng, Fengminggu; alt. 954 m; 8.viii.2017; leg. Xia Bai; genitalia slide Nos DNAYLL18123, XMR18241.

##### Differential diagnosis.

The new species resembles *D.trapezoides* (Meyrick, 1935), but the flagellum has three cinereous bands towards apex, the forewing has an obscure blackish stripe at middle of fold and the ostium bursae located at middle on anterior 1/3 of sternum VIII in the female genitalia. In *D.trapezoides*, the flagellum has two dark fuscous bands towards apex, the forewing has pale yellowish spots suffused with a few dark scales above fold at 1/3 and 3/5 and the ostium bursae opens at left of sternum VIII in the female genitalia.

##### Description.

**Adult** (Fig. 4): Wingspan 8.0 mm in holotype, 8.0–9.0 mm in paratypes. Vertex snow white to greyish-white, frons and occiput pale to smoky white. Antenna with scape snow white, without pecten; flagellum with alternate white and cinereous annulations, with three narrow cinereous bands towards apex. Labial palpus spatulate; yellowish-white on inner surface, black on outer surface, except yellowish-white at end of third palpomere. Thorax and tegula greyish-white, dusted with black. Forewing ground colour white, irrorate with blackish-brown and greyish scales, edged with bright ochreous yellow scales along termen and markings; patterned with black markings: costa with a wedge-shaped spot at base, a semicircular spot at 2/5, a larger semicircular patch from 3/5 to 4/5, diffused greyish smudges amongst spots; cell with a ribbon-like spot at distal 1/4, tending to coalesce with semicircular costal patch; fold with an obscure stripe at middle, obliquely inwards towards dorsum, surrounding suffusion of ochreous yellow; an interrupted terminal line around apex then along termen to tornus; cilia grey, with individual scales dark-tipped. Hind-wing and cilia grey. Legs yellowish-white, tibia black on outer surface, tarsus black on dorsal surface, except for end of each tarsomere.

***Male genitalia*** (Fig. 10). Uncus lobes fused into a terminally concave shield, bearing stout hair on caudal margin. Subscaphium an elongate band. Tegumen somewhat broad, with a hummocky process on left and caudal margins, respectively. Vinculum narrowed, sinuate, with a setose lobe at middle. Saccus not developed. A complicated, irregular, sclerotised modification attached to vinculum anteriorly, possibly part of segments VII and VIII; comprising of a Y-shaped sclerotisation and a receptacle-shaped sclerite that is enlarged and auricular at left. Sternum VIII articulated with vinculum at left, fused with left valva dorso-basally; oblong, folded, bottle-shaped, rounded and bearing long hairs apically, equipped with a spindle-shaped setose ridge beyond middle. Valvae strongly asymmetrical. Left valva battle axe-shaped, with a small finger-like lobe and a setose globular lobe at base; apodeme distinct, two. Right valva small, complicated: basal part broad, C-shaped; with a ribbon-shaped sclerite articulated with juxta; distal part bilobate into one large and one small lobe, both lobes slender, S-shaped, with an oval, setose apex. Juxta elliptical. Aedeagus a curved horn, tapered from base to a hook-like end; cornutus absent.

***Female genitalia*** (Fig. 11). Oviscapt reduced; anal papillae a pair of short, mastoid lobes. Posterior apophyses short, equalling the length of sternum VIII. Anterior apophyses slightly longer and stouter than posterior apophyses. Tergum VIII short, rectangular, intricately wrinkled antero-laterally, bearing short setae on posterior margin. Sternum VIII somewhat rounded, intricately wrinkled, bearing dense short setae on posterior margin. Ostium bursae located at middle on anterior 1/3 of sternum VIII. Antrum funnel-shaped. Ductus bursae slender, short. Corpus bursae irregularly elongate, without signum.

##### Distribution.

China (Hainan).

##### Etymology.

The specific name is derived from the Latin word *securiformis*, referring to the battle-axe-shaped left valva.

##### DNA barcode.

DNA sequencing resulted in a barcode of 604 bp from two paratypes: MZ711363/ DRYAD003-21 and MZ711364/ DRYAD004-21. The minimum distance (Table 1) to the nearest neighbour, *D.hirtiglobosa* sp. nov., is 6.06%.

### ﻿Checklist

Until this study, a total of 49 species have been described worldwide as identified in the checklist in Table 2. It is a taxonomic summary of the published works, contains type localities, depositories of types, distribution and available sources of figures for adults or genitalia that were given by previous researchers.

## Supplementary Material

XML Treatment for Dryadaula

XML Treatment for Dryadaula auriformis

XML Treatment for Dryadaula flavostriata

XML Treatment for Dryadaula hirtiglobosa

XML Treatment for Dryadaula securiformis
